# Tools to Alleviate the Drug Resistance in *Mycobacterium tuberculosis*

**DOI:** 10.3390/molecules27206985

**Published:** 2022-10-17

**Authors:** Ali A. Rabaan, Abbas Al Mutair, Hawra Albayat, Jawaher Alotaibi, Tarek Sulaiman, Mohammed Aljeldah, Basim R. Al Shammari, Amal H. Alfaraj, Mona A. Al Fares, Sara Alwarthan, Abdulwahab Z. Binjomah, Mohammed S. Alzahrani, Hatem M. Alhani, Mohammed S. Almogbel, Abdulmonem A. Abuzaid, Ghaya Alqurainees, Fatimah Al Ibrahim, Ali H. Alhaddad, Mubarak Alfaresi, Nadira Al-baghli, Saad Alhumaid

**Affiliations:** 1 Molecular Diagnostic Laboratory, Johns Hopkins Aramco Healthcare, Dhahran 31311, Saudi Arabia; 2 College of Medicine, Alfaisal University, Riyadh 11533, Saudi Arabia; 3 Department of Public Health and Nutrition, The University of Haripur, Haripur 22610, Pakistan; 4 Research Center, Almoosa Specialist Hospital, Al-Ahsa 36342, Saudi Arabia; 5 College of Nursing, Princess Norah bint Abdulrahman University, Riyadh 11564, Saudi Arabia; 6 School of Nursing, Wollongong University, Wollongong, NSW 2522, Australia; 7 Nursing Department, Prince Sultan Military College of Health Sciences, Dhahran 33048, Saudi Arabia; 8 Infectious Disease Department, King Saud Medical City, Riyadh 7790, Saudi Arabia; 9 Infectious Diseases Unit, Department of Medicine, King Faisal Specialist Hospital and Research Center, Riyadh 11564, Saudi Arabia; 10 Infectious Diseases Section, Medical Specialties Department, King Fahad Medical City, Riyadh 12231, Saudi Arabia; 11 Department of Clinical Laboratory Sciences, College of Applied Medical Sciences, University of Hafr Al Batin, Hafr Al Batin 39831, Saudi Arabia; 12 Pediatric Department, Abqaiq General Hospital, First Eastern Health Cluster, Abqaiq 33261, Saudi Arabia; 13 Department of Internal Medicine, King Abdulaziz University Hospital, Jeddah 21589, Saudi Arabia; 14 Department of Internal Medicine, College of Medicine, Imam Abdulrahman Bin Faisal University, Dammam 34212, Saudi Arabia; 15 Microbiology Department, Riyadh Regional Laboratory and Blood Bank, Riyadh 12746, Saudi Arabia; 16 Department of Infectious Diseases, King Abdulaziz Medical City, Ministry of National Guard Health Affairs, Jeddah 21423, Saudi Arabia; 17 Infectious Disease Department, King Saud Bin Abdulaziz University for Health Sciences, Jeddah 21423, Saudi Arabia; 18 Department of Pediatric Infectious Disease, Maternity and Children Hospital, Dammam 31176, Saudi Arabia; 19 Department of Infection Control, Maternity and Children Hospital, Dammam 31176, Saudi Arabia; 20 Preventive Medicine and Infection Prevention and Control Department, Directorate of Ministry of Health, Dammam 32245, Saudi Arabia; 21 Department of Medical Laboratory Sciences, College of Applied Medical Sciences, University of Hail, Hail 4030, Saudi Arabia; 22 Medical Microbiology Department, Security Forces Hospital Programme, Dammam 32314, Saudi Arabia; 23 Pediatrics Department, Prince Mohammed Bin Abdulaziz Hospital, Ministry of National Guard Health Affairs, Medina 41511, Saudi Arabia; 24 Infectious Disease Division, Department of Internal Medicine, Dammam Medical Complex, Dammam 32245, Saudi Arabia; 25 Assistant Agency for Hospital Affairs, Ministry of Health, Riyadh 12382, Saudi Arabia; 26 Department of Pathology and Laboratory Medicine, Sheikh Khalifa General Hospital, Umm Al Quwain 499, United Arab Emirates; 27 Department of Pathology, College of Medicine, Mohammed Bin Rashid University of Medicine and Health Sciences, Dubai 505055, United Arab Emirates; 28 Directorate of Public Health, Dammam Network, Eastern Health Cluster, Dammam 31444, Saudi Arabia; 29 Administration of Pharmaceutical Care, Al-Ahsa Health Cluster, Ministry of Health, Al-Ahsa 31982, Saudi Arabia

**Keywords:** *Mycobacterium tuberculosis*, drug resistance, mutations, antibiotics

## Abstract

Mycobacterium tuberculosis (*Mtb*), an acid-fast bacillus that causes Tuberculosis (TB), is a pathogen that caused 1.5 million deaths in 2020. As per WHO estimates, another 4.1 million people are suffering from latent TB, either asymptomatic or not diagnosed, and the frequency of drug resistance is increasing due to intrinsically linked factors from both host and bacterium. For instance, poor access to TB diagnosis and reduced treatment in the era of the COVID-19 pandemic has resulted in more TB deaths and an 18% reduction in newly diagnosed cases of TB. Additionally, the detection of *Mtb* isolates exhibiting resistance to multiple drugs (MDR, XDR, and TDR) has complicated the scenario in the pathogen’s favour. Moreover, the conventional methods to detect drug resistance may miss mutations, making it challenging to decide on the treatment regimen. However, owing to collaborative initiatives, the last two decades have witnessed several advancements in both the detection methods and drug discovery against drug-resistant isolates. The majority of them belong to nucleic acid detection techniques. In this review, we highlight and summarize the molecular mechanism underlying drug resistance in *Mtb*, the recent advancements in resistance detection methods, and the newer drugs used against drug-resistant TB.

## 1. Introduction

*Mycobacterium tuberculosis* (*Mtb*) causes the largest number of deaths by infectious diseases across the globe. Mtb is an ancient pathogen whose DNA has been recovered from a pre-Columbian mummy [1,2]. Over time, genome sequencing has revealed the co-evolution of this pathogen and the host defence system [3,4]. According to the Global Tuberculosis Report for 2021, 1.5 million people died from TB, of which 0.214 million were HIV positive [5,6,7]. In 2020, a total of 157,903 people with drug-resistant tuberculosis (TB) infections were reported, 132,222 of them were rifampicin-resistant cases or MDR-TB patients, and 25,681 were XDR-TB cases [5]. Given this context, we are forced to look for new treatments to combat Mtb infection, or it will be beyond our control [5,6,7].

In 2015, the United Nations (UN) and the member states of the World Health Organization (WHO) proposed a goal of eradicating the TB epidemic by 2030 and promoting the WHO strategy to end TB [8]. The End TB strategy aims to free the world of TB, with no more deaths and suffering caused by TB [8]. As per this strategy, intermediate milestones have been set for 2020, 2025, and 2030 to reduce the number of TB deaths by 95% and the number of new TB cases by 90%.

The co-occurrence of HIV infection in TB patients substantially increases the risk of TB reactivation [5,9]. In the absence of HIV co-infection, only about 10% of individuals exposed to *Mtb* develop active TB, which exceeds 10% in the case of HIV-co infection. In 2020, around 214,000 people who succumbed to TB had HIV co-infection [9]. The diagnosis and treatment of HIV-TB co-infection is another challenge and puts a tremendous burden on the healthcare system (World Health Organization Global Tuberculosis Report 2021) [5].

## 2. Drug Resistance in *Mtb*

Irrespective of the implication of a structured treatment regimen and effective therapeutic options for the past 70 years [10,11], the annual death rate worldwide due to TB remains above 1.5 million (https://www.who.int/teams/global-tuberculosis-programme/tb-reports, accessed on 10 July 2022). Most of these deaths occur in the reproductive and economically active age range of 15–50 years [12]. The factors contributing to increased chances of TB occurrence in different parts of the world are HIV co-infection, co-morbid conditions such as diabetes, disproportionately low access of populations in low-resource settings, and reduced socioeconomic standards [13]. One of the most common types of resistance is mono-drug resistance. However, poly-drug resistance is considered more severe than mono-drug resistance and must be addressed with utmost urgency [14].

By definition, multidrug resistance (MDR) refers to the resistance to first-line drugs, isoniazid and rifampicin [15]. On average, about 5% of newly diagnosed TB cases belong to the MDR category. The *Mtb* strains that display resistance against anti-TB drugs viz: isoniazid and rifampin, plus any fluoroquinolone drug, and at least one of the second-line drugs, are termed the extensive drug-resistant (XDR) strain [16]. The *Mtb* strains that display the MDR phenotype and rifampicin resistance and resistance to fluoroquinolones are termed the pre-XDR strains. A more dangerous form of TB, termed total drug-resistant (TDR), was identified as resistant against all first- and second-line drugs used for TB treatment [17,18]. About 9% of MDR TB develop into XDR TB, while 2% turn into TDR TB (https://www.who.int/news/item/12-05-2016-rapid-diagnostic-test-and-shorter-cheaper-treatment-signal-new-hope-for-multidrug-resistant-tuberculosis-patients, accessed on 8 September 2022). However, this may be an underestimation of the actual number since few people in endemic countries have access to proper diagnostic facilities for detecting drug-resistant TB. In addition, the existing diagnostic centres may not be using the most advanced and updated diagnostic techniques, which may lead to false negative results [19]. Therefore, there is an urgent need for better preventive measures, newer diagnostic methods, and advanced therapeutic approaches to curb the transmission rate of these drug-resistant strains worldwide.

### 2.1. Genetic and Molecular Components Underlying AMR in Mtb

#### 2.1.1. Intrinsic Drug Resistance

Numerous molecular mechanisms have evolved in *Mtb* that has endowed the pathogen with the ability to tolerate the cytotoxic effects of antimicrobial compounds, leading to intrinsic drug resistance [20,21]. The inherent drug resistance poses limitations over the disease treatment against conventional and newly discovered antimicrobials [22]. The mechanism responsible for intrinsic drug resistance in *Mtb* is described in the following section.

##### Spontaneous Mutation

The spontaneous and random mutations occurring within different regions of the bacterial chromosome may lead to the development of resistance against specific antimicrobial agents [23]. Although the rate of these mutations is prolonged, their occurrence early in the multiplication phase of the bacteria may lead to a clone of the bacteria that displays drug resistance. In the case of INH treatment, the spontaneous mutation rate was found to be 2.6 × 10^8^, while for rifampicin, it was 2.2 × 10^10^. The mutation rate against both drugs was found to be 10^12^, while for more than two drugs, it was observed to be 10^20^. This persistent state is due to the ability of the pathogen to remain in a non-replicating stage within the infected host cells in the presence of a hostile environment [24,25]. This stage accounts for the intrinsic and natural resistance of the pathogen against various classes of antimicrobial drugs [26,27,28].

##### Cell Wall Impermeability

Compared to other bacteria, the cell wall of *Mtb* displays reduced permeability toward various antimicrobial agents [29]. The movement of small hydrophobic molecules occurs quickly through the *Mtb* cell wall, while that of hydrophilic molecules is mediated by water-filled channels, termed porins [27]. Several other adaptations have enhanced the tolerance of *Mtb* towards antimicrobial agents by blocking or reducing their passage into the cells. These adaptations confer *Mtb* intrinsic resistance against several classes of chemotherapeutic agents [20].

##### Efflux Pumps

Efflux pumps are cell membrane proteins that aid in the expulsion of drug molecules from bacterial cells [20,30]. These efflux pumps make *Mtb* intrinsically resistant to many anti-tubercular drugs such as aminoglycosides, tetracyclines, and fluoroquinolones. In addition, these efflux pumps also play an essential role in the physiology, metabolism, and cell signalling process [31]. The efflux pumps in *Mtb* can be classified into different superfamilies such as (a) ATP binding cassette (ABC) superfamily, (b) Major Facilitator superfamily (MFS), (c) Small Multidrug Resistance (SMR) superfamily, (d) Resistance Nodulation Cell Division (RND) superfamily, (e) Multidrug and Toxic Compound Extrusion (MATE) superfamily [31].

Out of these five transporters, the ABC superfamily constitutes the primary transporter that relies on ATP as an energy source. In contrast, the remaining ones represent the secondary transportation system that relies on the proton motive force to expel drugs from the *Mtb* cells [32].

About 12 ABC transporters have been detected in the *Mtb* genome, and the corresponding genes constitute about 25% of the entire genome of the pathogen. Additionally, about 20 MFS and 15 RND transporters have been detected in *Mtb* isolates. MATE transporters have not been reported in *Mtb* yet, although they represent a common mechanism of drug resistance in Gram-negative pathogens [33]. Mycobacterial Membrane Protein Large (MmpL) is a subclass of RND transporters involved in lipid transport across the cell wall. The exported lipids with unusual structures play essential roles in the physiology and virulence of the pathogen. MmpS, Mycobacterial Membrane Protein Small, is a membrane fusion protein. Recent studies have suggested the involvement of MmpL and MmpS5 in drug resistance in *Mtb* [34].

The treatment of clinical strains with antibiotics has been shown to upregulate the expression of efflux pumps. For instance, upon exposure to isoniazid, ethambutol, and streptomycin, overexpression of MFS transporters has been reported [35]. Additionally, mutations in the genes encoding for these efflux pumps have also been linked to drug resistance [36,37,38,39].

##### Drug Modification and Inactivation

One of the most studied mechanisms of drug resistance in *Mtb* is the modification and inactivation of drugs. Many classes of enzymes encoded by the M. tb genome play an essential role in modifying or inactivating different types of antibiotics [40]. Penicillin is a class of β-lactam antibiotics that attack the transpeptidase group of enzymes and are involved in the cell wall synthesis of pathogens. Penicillin Binding Proteins (PBPs) are a group of transpeptidase enzymes involved in cross-linking peptidoglycan and play an essential role in cell wall synthesis. Many bacterial pathogens, including *Mtb*, have shown the production of a β-lactamase enzyme with the ability to hydrolyse the β lactam ring in penicillin and similar antibiotics, rendering them ineffective. *Mtb* has produced the following β-lactamases, BlaA, BlaC, BlaE, and type 2b [20]. In addition, the genome sequencing of *Mtb* has revealed the presence of four transpeptidases, all of which can bind to penicillin and similar antibiotics [20,40]. The BlaC gene in the *Mtb* genome codes for an extended-spectrum beta-lactamase that displays stronger penicillinase and cephalosporinase activity and a weaker carbapenemase activity [41].

In addition to the β-lactamases, the reduced permeability of cell walls to β-lactam antibiotics also results in virtually zero efficacy against *Mtb* [42]. The commonly used antibiotics against drug-resistant TB are carbapenems, such as meropenems and imipenem, which are used in combination with clavulanic acid. The carbapenems are also β-lactam antibiotics, but their hydrolysis by β-lactamase occurs more slowly. Its usage, along with clavulanic acid, an inhibitor of β-lactamase, makes this combination resistant to β-lactamases and highly effective against β-lactamase producing *Mtb* strains. Other forms of chemical modification of antimicrobial agents include methylation and acetylation, which prevent their binding to the intended molecular targets [20,40,42]. Another class of antibiotics used to treat drug-resistant TB are aminoglycosides. The major groups of enzymes modifying the aminoglycosides, namely, acetyltransferases and phosphotransferases, are encoded by *Mtb* chromosomes [43]. Among these two enzymes, N acetyltransferase has been characterized the most and has shown the ability to acetylate all the known aminoglycosides, including neomycin, gentamycin, kanamycin, amikacin, and tobramycin [44]. The best example of aminoglycoside inactivation is acetylation by enhancing intracellular survival proteins encoded by the *Eis* gene. The Eis protein can inactivate second-line aminoglycosides, kanamycin, amikacin, and capreomycin, injectable drugs for TB treatment [45,46,47]. Mutations in the promoter region of Eis protein may lead to its overexpression, which in turn confers low-level resistance [47,48,49]. In addition to the acetylation function, Eis protein confers protection to *Mtb* from host immune responses and enhances its survival in macrophages. This highlights the parallel evolution of virulence factors and antibiotic resistance [45,46,47].

##### Modification of Drug Targets

Another mechanism of developing intrinsic resistance to antibiotics in *Mtb* is the modification of their molecular targets, which hinders the binding of the antibiotics to their intended molecular targets. In *Mtb*, such target modifications have been shown to induce resistance against macrolides, streptomycin, and lincosamides [20,40,50]. These antibiotics bind within the larger subunit of bacterial rRNA and do not allow the translocation of the peptide–tRNA complexes. This further leads to the inhibition of protein synthesis and, thus, *Mtb* growth.

Another example of drug target modification is mutations in the *erm* gene. The erm gene encodes methyltransferase protein, which causes erythromycin resistance through the methylation of the erythromycin binding site in 23 s rRNA [20,50]. Another gene, *mfpA*, encodes for the pentapeptide repeat proteins, which protect against growth inhibition by quinolones resistance against viomycin and capreomycin upon binding to DNA gyrase. It has been shown to occur due to the loss of rRNA methyltransferase encoded by the *tlyA* gene [20,50].

Some *Mtb* proteins mimic the molecular targets of a few antibiotics that nullify their bactericidal activity. The best example is the resistance to fluoroquinolones. These classes of antibiotics inhibit replication and transcription, as well as repair of DNA by binding to DNA gyrase, preventing the sealing of fragmented DNA, leading to its degradation and, eventually, cell death. An *Mtb* protein termed MfpA (Mycobacterium Fluoroquinolone Resistance Protein A) resembles DNA in terms of size, shape, and topological properties. This similarity to the fluoroquinolone target causes DNA gyrase to bind to MfpA, preventing fluoroquinolone from binding to DNA gyrase [51,52].

**Figure 1:**
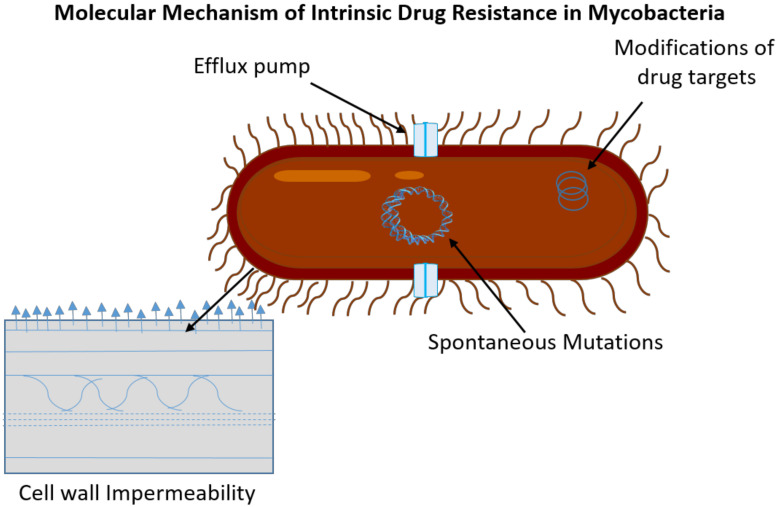
Diagrammatic illustration of the intrinsic mechanism of *Mtb* for drug resistance.

Various mechanisms underlying the intrinsic drug resistance in *Mtb* are summarized in Figure 1.

#### 2.1.2. Acquired Drug Resistance

The acquisition of resistance usually occurs due to selection pressure exerted by long-term exposure to antibiotics and non-compliance with antibiotic therapy. These resistant phenotypes are disseminated through either mutations or a horizontal mode of gene transfer via plasmids, transposons, and bacteriophages. Therefore, for the acquisition of resistance against antimicrobial drugs, the concentration of the drug determines the mutational pattern leading to the resistant phenotype [53,54]. At the suboptimal level of these drugs, mutations still occur while damaging the overall fitness of the bacteria, leading to reduced virulence and hence survival. In some commonly transmitted *Mtb* strains, low-cost or no-cost mutations have been shown to occur, but they still exhibit an enhanced level of drug resistance [53,54,55]. The reduced fitness occurring due to these mutations might be balanced through compensatory mutations that restore wellness while still retaining drug resistance. The term “compensatory mutations” refers to the secondary mutations that reduce or nullify the derogatory effect of the primary mutation. These secondary mutations may occur within the intra or extragenic locus ^39^. An example of the compensatory mutation in *Mtb* is the mutation in the gene *ahpC*, causing the overexpression of the enzyme alkyl hydroperoxide reductase. The overexpression of this enzyme compensates for the impaired fitness of *Mtb* due to primary mutations leading to isoniazid resistance [53,54,55].

##### Epistasis

Epistasis is the mechanism in which the effect of one mutation on the bacterial phenotype varies depending on the occurrence of another mutation [56]. Recent reports suggested the contribution of epistasis in shaping the evolution of antibiotic resistance [57,58,59,60]. However, most of these reports have studied the role of epistasis in the context of mono-drug resistance. In the context of the global threat imposed by the MDR strains, it is crucial to understand the underlying factors contributing to the emergence of the MDR phenotype. The epistatic interaction between different mutations imparting resistance to multiple drugs has been shown recently.

In a recent study, *M. smegmatis* was studied to depict the nature of the epistatic interactions between mutations imparting resistance to two drugs (rifampicin and ofloxacin). The mutants showing resistance to these drugs strongly correlated with mutations in the genes *rpoB* and *gyrA. gyrA* codes for a subunit of DNA gyrase that functions to introduce negative supercoiling in dsDNA [61], while *rpoB* codes for a segment of RNA polymerase involved in DNA transcription [62]. To begin with, the fitness cost of the mutants displaying resistance to either of the two drugs was determined from a series of resistant clones. It was observed in many *M. smegmatis* mutants showing resistance to these drugs that mutations imparting resistance to one of the drugs nullified the fitness cost associated with mutations imparting resistance to the second drug or vice versa. The data showed that *gyrA* D94G mutants co-related with the increased fitness in all the mutants showing double mutations. This phenotype was independent of the type of *rpoB* mutation.

Additionally, the relative fitness of these mutants depicting double resistance was higher than those showing resistance to one of the drugs, indicating the presence of sign epistasis. Sign epistasis refers to a condition in which the deleterious effects of a mutation can become beneficial in the presence of a second mutation [57]. In this study, 6 of the 17 double mutants showed the presence of sign epistasis, and the epistatic impacts were found to be allele-specific. In the case of MDR TB strains, the accumulation of different mutations conferring resistance to multiple drugs mitigates the fitness costs associated with individual mutations alone. These strains showed enhanced relative fitness compared to individual drug-resistant mutants [57].

**Figure 2:**
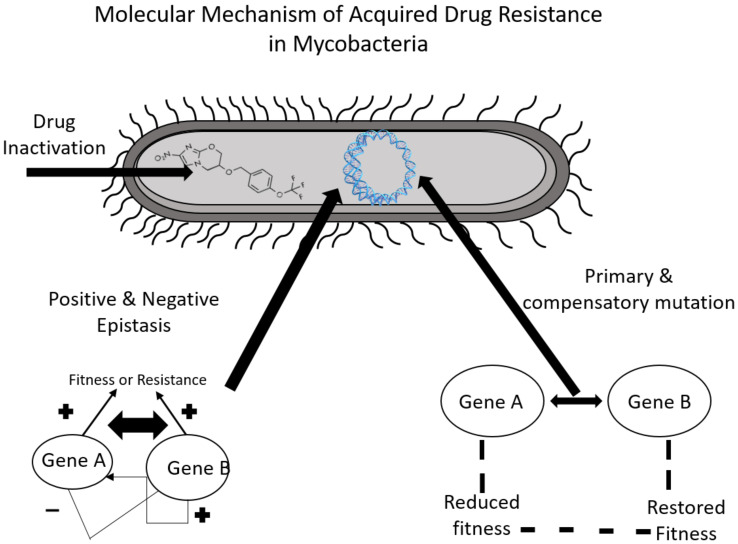
The mechanism for acquired drug resistance in *Mtb*.

The factors underlying the acquired drug resistance in *Mtb* are summarised in Figure 2. The genetic mutations associated with resistance to first- and second-line TB drugs are detailed in Table 1 and Table 2.

## 3. Recent Advancements in Methods to Detect Drug-Resistant *Mtb*

### 3.1. Conventional Phenotypic Methods

#### 3.1.1. Manual Culturing

Traditional culturing techniques using solid culture media such as Lowenstein Jensen media or various media types of Middlebrook such as 7H9, 7H10, and 7H11 have been widely used. Although these media are relatively cheaper and can be made manually, they lack standardization. Therefore, the antibiotic susceptibility tests (AST) performed using these media lack reproducibility and may vary depending on the culture and media types used.

#### 3.1.2. Semi-Automated Culturing

BACTEC Radiometric Method (BACTEC-460)

The semi-automated culturing system includes the Bactec 460 radiometric method (BD, USA). It is a 7H9 liquid culture media enriched with C14-labelled palmitic acid as the only source of carbon. Upon consumption of the carbon source, *Mtb* releases 14C-labelled CO2, which was detected by the Bactec instrument and used as a growth index (GI). The growth index of the untreated *Mtb* is compared to that of *Mtb* treated with antimicrobial drugs. This method is widely used to assess the antibiotic susceptibility of *Mtb* to first- and second-line TB drugs. Although this method is relatively faster as it requires only 5–10 days, using radioactive isotopes makes it expensive, and it is risky to handle and dispose of the radioactive waste generated using this method [122,123].

#### 3.1.3. Automated Liquid Culture System

The semi-automated culture system has been replaced by the wholly automated liquid culture system, which includes the BACTEC *Mtb* growth indicator tube (MGIT-960; BD, MD, USA), the MB/BacT culture system (Biomerieux, France), and the ESP culture system II (AccuMed International, USA).

These methods allow for faster growth of cultures of 14–21 days [124,125,126] and detect the CO_2_ production or O_2_ consumption using a fluorometer or a colourimeter. The best results, however, were obtained using a combination of solid and liquid culture media [126].

##### BacT/Alert 3D or MB/BacT Culture System

This is a non-radiometric and fully automated continuous culture method. It is based on detecting carbon dioxide gas (CO_2_) using a colourimetric system connected to an advanced computation system that quantifies the amount of gas released. The actively metabolising *Mtb*, upon utilization of carbon source, releases CO_2_, which changes the colour of the liquid emulsion sensor (LES), a colour indicator at the bottom of the vial, changing the colour from blue to yellow. These colour changes are detected through reflectometry placed in each incubation chamber of the system. Although this method is faster than the manual method of culturing and widely accepted for TB diagnosis, it may be prone to be contaminated, has a longer turnaround time, and is expensive due to the sophisticated machine [127,128].

##### Mycobacterial Growth Indicator Tube (MGIT) Method

This method is based on the fluorescence-based detection of oxygen depletion due to *Mtb* growth in the culture vial upon UV illumination. This oxygen depletion is directly proportional to the fluorescence intensity, which is termed as growth unit (GU). The GU of control untreated *Mtb* culture reaches a value of 400 within 4–13 days. The GU value of the control *Mtb* is then compared to the GU value of the drug-treated *Mtb* culture, which is found to be more than 100 and is termed as the culture displaying resistance to that particular drug [123,129]. Many reports have shown this method’s ability to rapidly diagnose drug-resistant *Mtb* toward first- and second-line TB drugs. Compared to traditional methods, the MGIT method performs equally well but is prone to contamination due to the use of liquid culture media.

##### VersaTREK System/ESP II System

The VersaTREK or ESP II system is a non-radiometric and fully automated method that provides the benefit of continuous monitoring. In this method, the growth measurement of *Mtb* is based upon changes in the pressure inside the culture vial due to either production or consumption of gases owing to bacterial growth. Automated kits for testing drug sensitivity against isoniazid, rifampicin, ethambutol, and pyrazinamide are available, but they are cumbersome and require 14–30 days [130,131].

**Figure 3:**
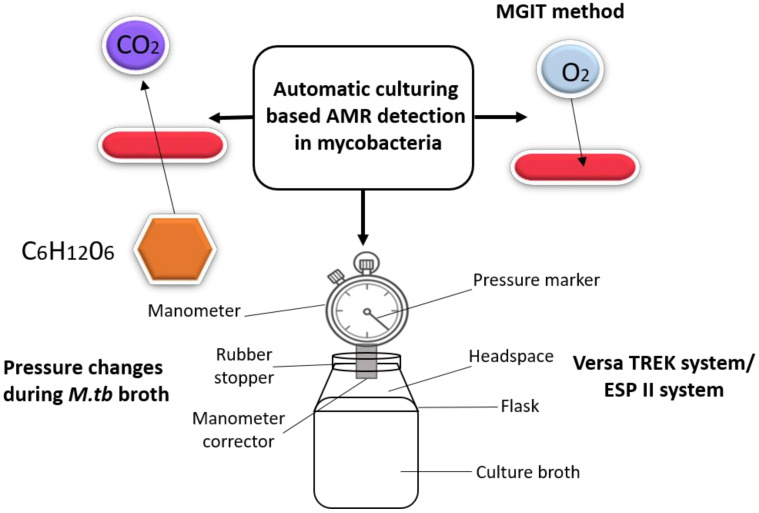
Recent advancements in automated culturing methods for drug resistance detection in *Mtb*.

The advancements in automated culturing methods for detecting drug resistance are summarised in Figure 3.

##### Microscopic Observation Drug Susceptibility Assay (MODS)

This method is based on the microscopic detection of cord factor formation, a characteristic of *Mtb*. The accuracy of detecting drug sensitivity is highly comparable to standard reference methods, i.e., 97% for INH, 100% for RIF, and 100% for fluoroquinolones [132]. However, lower accuracy rates are displayed in the cases of ethambutol (95%) and streptomycin (92%) [133]. This technique needs a maximum of 7 days to obtain the results for drug sensitivity.

#### 3.1.4. Colorimetric Redox Indicator Methods

These methods utilise the principle of colourimetric detection of the colour change of a redox indicatory dye present in the culture vial. The colour change of the redox indicator dye is proportional to the viable *Mtb* within the growth vial, which indicates the presence of drug resistance. In addition, of different antibiotics, the per cent viability of *Mtb* may or may not change, which eventually corresponds to the magnitude of the colour change of redox dye [33].

Two redox dyes that are mainly used are resazurin and MTT. Resazurin is a redox indicator dye currently sold under the name Alamar Blue. It is blue in colour in the oxidised state, but upon reduction by *Mtb* growth, it changes to pink colour. It has been used to detect drug-resistant *Mtb* against various anti-TB drugs. The overall accuracy of this method reaches up to 97% compared to the conventional agar-based method in the case of INH, RIF, EMB, and STR [129,134]. Another redox dye, MTT (tetrazolium bromide 3-(4,5-dimethyl-2-thiazolyl)-2,5-diphenyl-2 H-tetrazoliumbromid), is reduced by the microbial dehydrogenase group of enzymes that is a characteristic of living bacterial cells. It is a yellow-coloured dye that is reduced to purple-coloured insoluble formazan crystals by the metabolically active *Mtb*. The reduction of MTT can be measured spectrophotometrically following the solubilisation of the crystals with an organic solvent. The MIC values for each drug can be obtained within 1–2 weeks. It has been widely employed for detecting resistance to RIF and other medicines used for TB treatment with good accuracy [126,135].

#### 3.1.5. Mycobacteriophage-Based Methods

Mycobacteriophages are viruses that infect *Mtb* and replicate inside them. In the Pha B assay, the intracellular phages depicting the actual number of viable *Mtb* can be estimated by plaque formation assay. A rapidly growing mycobacterial species, *M. smegmatis,* can also be used to get rapid results [136,137]. The commercial FastPlaque TB kit has been used to detect rifampicin resistance in laboratory and clinical strains of *Mtb*. The time required to complete the process to get the results is about two days [137].

#### 3.1.6. Luciferase-Based Reporter Phage Method

This method is based upon the production of luminescence by live *Mtb* cells infected with reporter phages expressing luciferase enzyme. The light signal can be detected shortly post-infection, whose intensity depends on the phage dosage, level of luciferase expression, and intracellular ATP pool [44]. The *Mtb* strains showing susceptibility towards INH or RIF display a time-dependent decrease in luminescence, whereas the drug-resistant bacteria will go with the production of luminescence. These phage-based methods tend to possess higher sensitivity but reduced specificity due to variable factors. The average detection time of bacteria is seven days [137,138].

#### 3.1.7. Microcolony Method

The microcolony method detects the presence of *Mtb* microcolonies on a thin layer of solid culture media such as 7H11 agar using a microscope. This method can be applied to sputum samples obtained from MDR TB patients. Preliminary studies have supported the accurate detection of MDR TB within seven days [139].

#### 3.1.8. E-Test (AB BIODISK)

This method relies on paper strips impregnated with different concentrations of anti-TB drugs, thus creating a concentration gradient. The results can be obtained in 5–10 days but show a higher proportion of false positive results than automated culturing methods [140].

#### 3.1.9. TK Medium

TK medium is a culture media commercially developed to contain indicator dyes and used for the diagnosis and antibiotic sensitivity of *Mtb*. The actively metabolising *Mtb* changes the colour of the media from red to yellow, which is distinctly visible. It reduces the time for getting results to about three weeks, nearly half of the other conventional media, such as LJ media [141].

#### 3.1.10. Nitrate Reductase Assay

Live *Mtb* can reduce nitrate into nitrite, which can be detected using a chemical reagent added to the culture media. It uses traditional LJ media supplemented with anti-TB drugs and a source of nitrate. Viable *Mtb* can change the colour of the media in 7–14 days post-incubation. This method shows comparable specificity and sensitivity toward detecting resistance to INH, RIF, STR, and EMB compared to traditional methods [142,143,144,145].

### 3.2. Biochemical Method

Mycolic Acid Index Susceptibility Testing

It is a modified method based on mycolic acid analysis by HPLC, which uses coumarin to derive fluorescence from mycolic acid. The sensitivity against a drug is estimated by measuring the area under the chromatography peaks of mycolic acid, which correlates well with the log of colony-forming units (CFU) per ml. The method can be completed in 5–10 days [146]. This method has been used successfully to evaluate the susceptibility of *Mtb* to isoniazid and rifampicin [147].

### 3.3. Genotypic Methods

**Figure 4:**
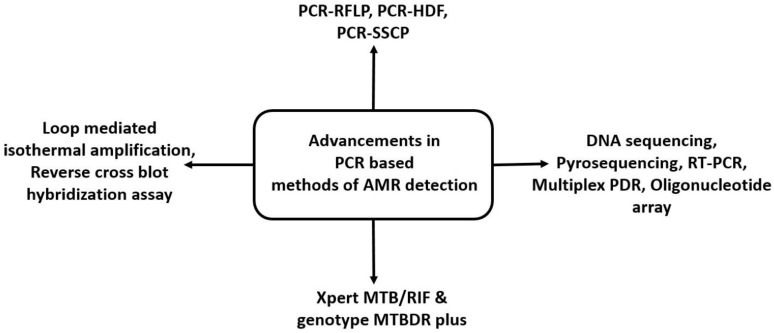
Nucleic acid detection method for drug resistance in *M.tb* based on PCR technique.

Various molecular methods that detect the nucleic acid sequence based on PCR-based amplification are described below and summarised in Figure 4.

#### 3.3.1. DNA Sequencing

DNA sequencing is a molecular method that remains a milestone in detecting drug resistance in *Mtb* strains, unravelling the entire sequence of the nucleotides contained within the gene of interest. The time taken to complete it is merely 10–12 h, much faster than the culture-based methods. However, the major drawback is that some of the mutations may be silent and unrelated to drug resistance.

However, performing DNA sequencing to detect drug resistance-conferring mutations is not as easy as species identification. For instance, mutations in multiple genes may play a role in mono-drug-resistant strains, as in the case of INH resistance. This indicates obtaining all the data regarding all the mutations associated with a single drug. In a single *Mtb* strain, either different sequencing methods need to be applied, or the entire genome of the isolate needs to be sequenced. However, in the case of specific genes, such as the analysis of *rpoB* mutations in the case of RIF resistance, it remains the method of choice due to the ease of performing and less time consumption [148,149,150].

#### 3.3.2. Pyrosequencing

Pyrosequencing is mainly used to identify the single nucleotide polymorphism (SNP) associated with drug resistance and to perform sequencing of short reads [151,152].

In a study performed in Sweden, pyrosequencing was utilised to simultaneously detect mutations underlying resistance to isoniazid, rifampicin, ethambutol, ofloxacin, amikacin, kanamycin, and capreomycin. This method depicted a great degree of specificity and sensitivity, 100% and 94.6%, respectively, to detect MDR TB. It also showed higher specificity and sensitivity, 99.3% and 86.9%, to see XDR TB. It also helped to detect specific mutations in the *rpoB* gene in 96.7% of rifampicin-resistant *Mtb*, the *katG* gene in 64% of isoniazid-resistant *Mtb*, and the *gyrA* gene in 70% of ofloxacin-resistant-*Mtb* isolates [153].

Pyrosequencing is more popular than traditional sequencing techniques due to the reduced time required for process completion (6 h). The major drawback remains the limited sequencing of a DNA sequence up to 20–50 nucleotides long. In the case of a more extended DNA sequence, traditional DNA sequencing techniques are preferred [151].

#### 3.3.3. Oligonucleotide Microarray

This technique allows the detection of various gene sequences simultaneously in a single reaction using primers specific for the conserved sequences or specific gene segments suspected of carrying mutations conferring resistance to a particular drug. A TB biochip developed in Europe has been utilised to detect mutations associated with rifampicin resistance in *Mtb*. Using a combination of microarray and routine antibiotic susceptibility testing, the method detected a rifampicin sensitivity profile of 80% of isolates [154].

#### 3.3.4. Whole Genome and Next Generation Sequencing

Whole Genome Sequencing using the Next Generation Sequencing platforms, Illumina (for shorter reads) or nanopore (for longer reads), represents a revolution for estimation of the genotype, investigating the infection outbreak, and determining the single nucleotide polymorphism (SNPs) associated with the antimicrobial resistance. It involves massively parallel sequencing of targeted or non-targeted genomics segments, generating unique DNA fragments termed reads [155,156]. The emergence of drug resistance in *Mtb* strains is due to the genetic changes at the single nucleotide level, which may lead to the insertion or deletion of genes encoding molecular targets of various drugs such as efflux pumps, metabolic pathways, etc. Therefore, detecting these genomic changes using a high throughput methodology offers rapid and better management of drug-resistant TB cases [157].

### 3.4. PCR-Based Methods

#### 3.4.1. Multiplex Allele-Specific PCR (MAS-PCR)

MAS-PCR was first applied to detect ethambutol resistance in *Mtb* isolates and later applied successfully to detect isoniazid and rifampicin resistance. It works on the same principle as the traditional PCR technique. However, it uses two primers against the flanking regions surrounding the gene of interest and the primers specific to the alleles present in the inner wild-type gene fragment. Any nucleotide alteration at the 3′ end of the allele-specific primer leads to the inability of the polymerase to extend the primer and, thus, the non-amplification of the fragment. This method can be completed in 6–7 h and displays excellent sensitivity and specificity toward detecting resistance against first- and second-line TB drugs [158,159,160].

#### 3.4.2. PCR SSCP (PCR-Single Strand Conformation Polymorphism)

The SSCP method can detect numerous variations of the single-stranded DNA sequence. It is based on the observation that an ssDNA adopts a specific conformation unique to its nucleotide sequence. Therefore, even a single nucleotide variation in the sequence leads to the alteration of its conformation. These conformational changes may alter the electrophoretic mobility of the sequence. SSCP has been used to detect resistance to isoniazid, rifampicin, streptomycin, and ciprofloxacin [161]. The results can be obtained in 10–14 h with an accuracy of 95% [162].

#### 3.4.3. PCR Hetero-Duplex Formation (PCR HDF)

PCR HDF was initially used to detect mutations in the rpoB gene associated with rifampicin resistance in *Mtb* strains. A hybrid DNA mixture is obtained by mixing DNA from a test isolate and a drug susceptible isolate. The presence of gene mutations produces a heteroduplex hybrid DNA in the mix, which eventually results in changes in the electrophoretic mobility compared to the homoduplex hybrid DNA observed in the case of wild-type DNA. This method requires approximately 6 h to produce the results [163].

#### 3.4.4. Solid-Phase Hybridisation Assay

In the solid-phase hybridisation method, the specific region associated with resistance is initially amplified using primers labelled with fluorescent or other dyes. The amplified product is then hybridised to the probe immobilised on a solid phase, and the hybridisation is detected using the colour or fluorescence generated during the hybridisation. In the case of any mutation in this specific region, the amplicon will not be able to hybridise to the probe complementary to the wild-type gene sequence. Still, it will hybridise with the probes that share the complementarity with the mutation in that region. The most widely used examples of assays that rely on this principle are Line Probe Assays (LiPAs) and DNA microarray-based biochips. LiPAs involve three stages (1) extracting DNA, (2) performing multiplex PCR, and (3) reverse solid phase hybridisation, requiring a total duration of 5 h.

Currently, three kit-based tests relying on this principle are promoted by the WHO for the detection of drug resistance in *Mtb* (1) GenoType *MTB*DR, (2) GenoType *MTB*DRplus, and (3) INNO-LiPA RiF TB. These tests detect gene mutations in the *MTB*C complex related to drug resistance in isolates obtained from smear-positive sputum samples [164]. It displays 82–100% sensitivities for detecting rifampicin resistance-associated mutations in *MTB*C members with specificities of 92–100% [165]. The *MTB*DR method can detect both isoniazid and rifampicin resistance simultaneously and successfully diagnose MDR TB [166,167,168]. Recently, a modified method, called GenoType*MTB*DRsI, was developed to detect resistance to second-line drugs such as fluoroquinolones, capreomycin, and amikacin in *MTB*C members and showed remarkable sensitivity [169].

The significant merits of this method are that it can be performed directly on smear-positive sputum samples, improved documentation of results, and the usage of advanced instrumentation. The disadvantage involves the requirement of various probes to span overlapping genetic regions, and the silent mutations may still give false resistance predictions. In addition, they require designated space and highly skilled personnel and are labour-intensive [166,167]. A recent study utilised DNA microarray to analyse clinical isolates for mutations in seven DNA sequences related to resistance to five anti-tubercular drugs: isoniazid, rifampicin, streptomycin, kanamycin, and ethambutol. A higher sensitivity of 90% was detected in the case of all five drugs [170]. The per cent specificity was over 90% in the case of rifampicin and ethambutol, but it was lower than 70% for isoniazid and kanamycin within a duration of 5–7 h [165].

#### 3.4.5. PCR Restriction Fragment Length Polymorphism (PCR-RFLP)

RFLP is a simple and reliable method that yields reproducible results in identifying drug-resistant *Mtb*. A study performed in Russia used PCR-RFLP to detect isoniazid resistance-associated mutations in the *katG* gene in 93.6% of the resistant strains [171]. In another study conducted in China, PCR-RFLP was used to detect *katG* mutations in 51% of the drug-resistant isolates [172]. Using this method, results can be obtained within 8–10 h post-extraction of DNA.

#### 3.4.6. Multiplex-PCR

Multiplex-PCR uses various primer pairs to detect multiple genes in a single sample simultaneously. It was used for the first time to detect point mutations in *katG* (AGC to ACC) and *inhA* (C-15-T) genes associated with resistance to isoniazid and ethionamide. Approximately 68% of the resistant *Mtb* isolates showed the presence of one or both of the mutations. This method can be modified further to enhance the accuracy of detecting the mutations associated with isoniazid resistance in clinical isolates of *Mtb*. It takes 7–8 h to obtain the results with this method after extracting DNA [173].

#### 3.4.7. PCR-Reverse Cross-Blot Hybridisation Assay

Reverse cross-blot hybridisation assays were first performed to analyse the genotypes of various drug-resistant *Mtb* strains [174]. The methods rely on oligonucleotide probes tailed with dTTP to capture the target DNA sequence efficiently. After blotting them onto a positively charged nylon, PCR products were allowed to hybridise. The hybridisation of PCR products is detected by incubating with a streptavidin-alkaline phosphatase complex and a chromogenic substrate. This technique detected mutations in *katG*, *inhA*, and *ahpC* associated with isoniazid resistance in 82% of the clinical isolates within 10–12 h. However, this method cannot see all types of phenotypic resistance related to isoniazid and rifampicin in clinical isolates obtained from MDR TB patients [175].

#### 3.4.8. Loop-Mediated Isothermal Amplification

The LAMP method works on a similar principle as that of the PCR with key differences, (1) the amplification of the target gene is performed at a constant temperature of 60–65 °C, and (2) Instead of Taq polymerase, DNA polymerase is used due to a more vital DNA strand displacement ability in addition to the routine DNA replication activity. A combination of four specific primers is used to identify approximately six distinct gene sequences on a target DNA, providing better specificity than conventional PCR reaction [176]. The number of PCR products generated using LAMP is also more significant compared to routine PCR techniques. The amplified product can be detected by fluorescence, photometry, or turbidity changes due to the precipitation of magnesium pyrophosphate. Although this method has been used very limitedly, it shows higher sensitivity in the case of smear-positive TB samples but lower for smear-negative TB samples [177].

#### 3.4.9. Xpert MTB/RIF

GeneXpert is a semi-automated technique using real-time PCR using molecular beacons to detect DNA sequences. It uses a multiplex reaction that contains different hybridisation probes that shows complementarity towards another segment within a single target gene and are labelled with other fluorescent dyes. While detecting mutations in rpoB, the gene using this technique, the whole part of 81 bp corresponding to the rifampicin resistance determining region (RRDR), is covered using multiple probes [178]. The method has shown 100% sensitivity for smear-positive TB samples and 71% in the case of smear-negative but culture-positive TB samples [179]. The pros of this method are the enhanced degree of detection sensitivity, shorter time for diagnosis (2 h) and treatment, and the requirement of minimal technical expertise. It can also be used in the case of raw sputum samples and extrapulmonary clinical samples. The demerits include a higher cost and the inability to detect isoniazid resistance.

### 3.5. Artificial Intelligence (AI) Based Detection Methods

Artificial intelligence-based machine learning methods such as support vector machine (SVM), logistic regression (LR), as well as random forest (RF) has been used to detect drug resistance [180,181,182].

In a study on 161 isolates, LR was used to assess the new genes related to resistance in *Mtb* isolates against seven drugs [181]. Another study in the United Kingdom applied the data from 1839 *Mtb* isolates to compare the classification models for the medications used to treat drug-resistant *Mtb*^165^. At the same time, RF was used on a diversified dataset obtained from various geographical locations on 1397 *Mtb* isolates [180].

In recent work, machine learning-based approaches were used to predict mutations in the genes *rpoB, katG, gyrA, pncA, gyrB*, and *inhA* associated with resistance to drugs such as rifampicin, pyrazinamide, fluoroquinolones, and isoniazid. The algorithms used to generate prediction models were naïve Bayes, SVM, artificial neural network (ANN), and k nearest neighbour. These models displayed accuracy of 85% for all the genes [183].

## 4. Current Approaches for the Treatment of Drug-Resistant TB

### 4.1. Bedaquiline

This is a novel diarylquinoline compound with specific activity against *Mtb* due to its inhibitory activity against the mitochondrial enzyme ATP synthase. The WHO recommends using bedaquiline to treat MDR and XDR TB in combination with existing drugs [184]. In a recent study, the outcomes of 428 MDR TB patients treated with bedaquiline-containing treatment regimens were reported from 15 countries [184]. The success rate was 77%, which is 10% higher than a study conducted in South Africa. In only 6% of patients, bedaquiline was withdrawn due to serious side effects, with only one death due to cardiovascular anomalies ^167^. Currently, it is being studied in the Nix-TB trial that evaluates the six months regimen of bedaquiline, pretomanid, and linezolid at a dose of 600 mg twice daily. If the patients are sputum positive at the end of 4 months, the regimen is repeated for another 3 months. The Nix TB trial showed that 86% of patients who completed this treatment regime did not display signs of disease relapse for the next 6 months of follow-up. Despite the death of four patients in the initial treatment stage, by the end of the fourth month, the culture conversion rate was 65%.

### 4.2. Delamanid

Delamanid belongs to the metronidazole class that inhibits mycolic acid synthesis. The WHO has recommended using the demand for the treatment of MDR and XDR TB in combination with three other drugs with proven efficacy.

Almost 700 patients have received treatment with delamanid, which shows favourable outcomes (World Health Organization, 2014). The success rate was 74% in phase 2 trial 204 [185], 81% in phase 2 trial 213, and 84% in a cohort-based study conducted in Latvia. The sputum conversion rates were achieved in 80% of the patients that underwent treatment [186].

### 4.3. Pretomanid

Pretomanid is a nitroimidazole developed for treating drug sensitive and MDR TB. In an advancing novel drugs trial aimed at reducing the treatment span, pretomanid is combined with moxifloxacin and pyrazinamide for two different duration intervals of 4 and 6 months [187].

### 4.4. Linezolid

Linezolid belongs to the oxazolidone class of antimicrobial agents that inhibits protein synthesis b interacting with the 70s bacterial ribosomes [188]. It has been shown to improve the treatment outcome of patients with drug-resistant TB [189,190]. The WHO has approved the usage of linezolid (600 mg) daily for rifampicin-resistant TB. However, due to structural homology with mitochondrial rRNA, linezolid usage is associated with severe side effects depending on the dose and the duration of the linezolid treatment [190,191].

### 4.5. Clofazimine

Clofazimine (CFZ) belongs to the riminophenazine class of antibiotics that display anti-tubercular activity [192,193]. CFZ appears to target the electron transport chain and the ion transporters [194,195]. It has shown potent activity against MDR TB in both in vitro and in vivo conditions [196,197]. Its MIC value against MDR *Mtb* strains ranges from 0.125 to 2.0 mg/L [198,199]. Therefore, CFZ has recently been added to the treatment regimen for MDR TB [200,201].

### 4.6. β-Glucan

Yeast-derived glucan particles (YDGP) are 2–4 μm in diameter and of a porous as well as polymeric nature. They are synthesised using yeast cell walls and consist of glucose monomers [202]. β-glucan functions as the Pathogen Associated Molecular Pattern (PAMP), which is recognised by Pattern Recognition Receptors, such as Toll-Like Receptors [203]. The particles synthesised using β-glucan show excellent efficiency in encapsulating nucleic acids, proteins, and small molecules [204,205,206,207,208].

β-glucan particles can be uptaken by the phagocytic cells via receptor-mediated endocytosis as they express β-glucan receptors. Therefore, they have been emerging as novel drug delivery tools. In a recent study, glucan particles derived from yeast were pre-loaded with rifabutin nanoparticles. The efficacy of these hybrid nanoparticles in inhibiting the intracellular *Mtb* within murine macrophages was investigated. It was observed that these hybrid NPs stimulate a wide range of innate immune response pathways, including ROS and RNS, autophagy, and cell death pathways within *Mtb*-infected murine macrophages. This formulation also enhanced the efficiency of rifabutin 2.5 times [209].

In a recent study, YDGP has been shown to modulate phagosome maturation and induce autophagy via the NOX-2 pathway. These results show the potential of YDGP in immunomodulation in addition to targeted drug delivery and also augment autophagy-mediated therapeutics [210].

## 5. Conclusions

Despite the continuous deaths due to *Mtb* worldwide, researchers are making extensive efforts to devise newer strategies to control the problem. There have been numerous advancements in detecting genetic mutations associated with drug resistance. These methods mainly focus on detecting the nucleotide sequence of genes and alterations in shape and conformation due to nucleotide changes. The advances in genome sequencing techniques have allowed the detection of even a single nucleotide and allele-specific variations. Additionally, several new drugs have been tested in pre-clinical and clinical trials, some of which have shown tremendous potential. However, the extensive use of these new drugs may also induce mutations, eventually leading to drug resistance. Therefore, judicial and intelligent usage of the new antimicrobial agents in combination with the existing regimen may prove beneficial in the long term and may reduce the rate of resistance generation against these drugs. Additionally, artificial intelligence-based algorithms have proven helpful in predicting the rate of drug resistance and assessing the best combination of medications for the treatment of drug-resistant TB.

## Figures and Tables

**Table 1:** Types of genetic mutations in mycobacteria leading to resistance against first-line drugs. * Represents found in Drug-resistant and/or Drug-sensitive strains.

Sr. No	Type of Drug Resistance	Genes Displaying Mutations	Type of Mutations	References
1.	Isoniazid	*inhA, katG, kasA, ahpC, ndh, furA*,	**Mutations in inhA** Point mutations in inhA codons S94A, codons:16 (I-T); 21 (I-T and I-V); 47 (I-T), 78 (V-A), 94 (S-T), 95 (I-P) M1L, K8N, I16T, I21T,V, I25T, I47T *,V78A, S94A, I95P, A190S, I194T *, R202G, E217D, T241M, T253A *, D256N, I258T,V, Y259H Point mutations in inhA promoter Regions -15(C-T), -16(A-G), -8(T-G/A), and -24(G-T) **Mutations in katG** Codon 138, 328, 315 (S-T), 463 (R-L) **Mutations in kasA** Codons 66 (G-A), 269 (G-A), 312 (G-A), 413 (C-A), D66N *, M77I *¸ R121K, L245R¸ G269S *, G312S *, S341 *¸ G387D, F413L **Mutations in APC** Promoter regions -57 C-T; -54 C-T; -52 C-T -51 G-A; -48 G-A; -47/-46 T-insertion, P2S, L3K, L4R, T5I, F10I, D33N, D73H *, E76K, L191K **Mutations in ndh** Codons 110 (T to A) & 268 (R to H), CGT to TGT change in codon 13, and GTG to GCG change in codon 18, R13C, V18A*, T110A, R268H, G313R * Mutations in furA S5P, c34 del, A14V, A46V *, L68F, C97Y	[63,64,65,66,67,68,69,70,71,72,73,74,75,76,77,78,79,80,81,82,83,84,85,86,87,88,89,90]
2.	Rifampicin	*rpoB*	**Rifampicin Resistance Determining Region** codons 507, 516, 526, 531, 533	[21,64,91,92,93]
3.	Pyrazinamide	*pncA*	Clustered mutations (amino acids 3–71, 61–85, and 132–142) codon 114 (T to M)	[94,95,96]
4.	Ethambutol	*embB, embC, embC*	codon 306 (A to G, A to C, G to A, and G to C); codons at 285 (F to L), 330 (F to V), and 630 position (T to I)	[97,98,99,100,101,102,103]

**Table 2:** Types of genetic mutations in mycobacteria leading to resistance against second-line drugs. * Represents found in Drug-resistant and/or Drug-sensitive strains.

Sr. No	Type of Drug Resistance	Genes Displaying Mutations	Type of Mutations	References
1.	Streptomycin	*rrs, rpsL, gidB*	C-T transition at positions 491, 512, and 516; A-C/T transversion at position 513; 903 (C to A/G) and 904 (A to G); codon 43 (AAG to AGG/ACG); codon 88 (AAG to AGG/CAG)	[104,105,106,107,108,109,110,111]
2.	Fluoroquinolones	*gyrA, gyrB*	D94G, D94Y, D94N, D94A, D94H, A90V, codon 95 (Ser95 > Thr95); G512Rcodons	[112,113,114]
3.	Aminoglycosides (Kanamycin & Amikacin)	*rrs*	A1400G, C1401A, and G1483T	[48,115]
4.	Ethionamide	*etaA (ethA)*	S94A c-15t in the promoter of inhA, M1R, A234D, C403G I9T, t703 del, R404L G11A, Q246STOP, G413D g32 del, A248D, c1254 del A20 ins, Y250ST0P, g1268 del, a65 del, cg754 ins, c1290 del, H22P, Q254P, gc1322,1323 del, Y32D, Q254ST0P, T453I, a110 del, g768 del *, Y461H, G43S, C, S266R, R463D, T44N, Q269ST0P, a1391 ins, D49A, Q271ST0P	[116,117,118,119,120,121]

## Data Availability

Not applicable.
